# Shallow carbon storage in ancient buried thermokarst in the South Kara Sea

**DOI:** 10.1038/s41598-018-32826-z

**Published:** 2018-09-25

**Authors:** Alexey Portnov, Jürgen Mienert, Monica Winsborrow, Karin Andreassen, Sunil Vadakkepuliyambatta, Peter Semenov, Valery Gataullin

**Affiliations:** 1 School of Earth Sciences, The Ohio State University, Columbus Ohio, USA; 2 CAGE - Centre for Arctic Gas Hydrate, Environment and Climate, Department of Geosciences, UiT The Arctic University of Norway, 9037 Tromsø, Norway; 3 FGBU VNIIOKEANGEOLOGIYA, Saint-Petersburg, Russia; 4 7159 Crofton Court, Reynoldsburg, OH 43068 USA

## Abstract

Geophysical data from the South Kara Sea reveal U-shaped erosional structures buried beneath the 50–250 m deep seafloor of the continental shelf across an area of ~32 000 km^2^. These structures are interpreted as thermokarst, formed in ancient yedoma terrains during Quaternary interglacial periods. Based on comparison to modern yedoma terrains, we suggest that these thermokarst features could have stored approximately 0.5 to 8 Gt carbon during past climate warmings. In the deeper parts of the South Kara Sea (>220 m water depth) the paleo thermokarst structures lie within the present day gas hydrate stability zone, with low bottom water temperatures −1.8 ^o^C) keeping the gas hydrate system in equilibrium. These thermokarst structures and their carbon reservoirs remain stable beneath a Quaternary sediment blanket, yet are potentially sensitive to future Arctic climate changes.

## Introduction

**Figure 1:**
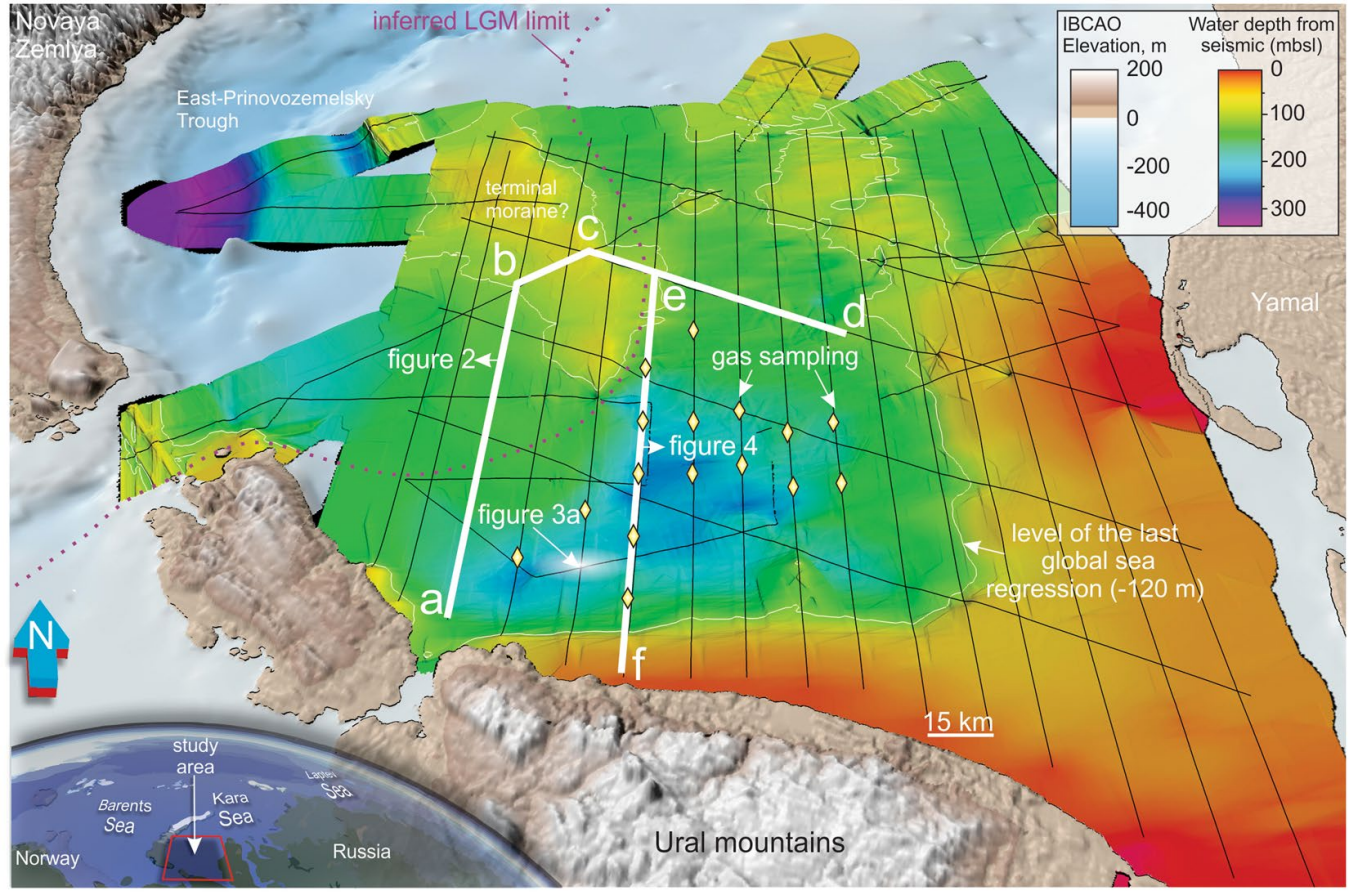
Bathymetry of the South Kara Sea in pale blue color scale (IBCAO v.3) and rainbow color scale (based on high-resolution seismic data). Inset shows the location of the study area (map was generated based on IBCAO v.3 bathymetry grid in Global Mapper v.18 software, http://www.bluemarblegeo.com). Thin black lines show high-resolution seismic grid used in the current study, white thick lines indicate location of seismic profiles, shown in Figs 2 and 4. Yellow diamond shapes show locations of bottom sediment gas-sampling stations used for gas hydrate stability zone modeling. Last Glacial Maximum eastward ice sheet extent^23,24^ is indicated with purple dashed line.

In the Arctic, modern on- and offshore permafrost covers several million square kilometers. This initially developed adjacent to the large Quaternary ice sheets during glacial periods when sea level regressions (≤120 m) repeatedly exposed the Arctic continental shelves to frigid subaerial conditions^1^. During such periods, annual surface temperatures dropped 10–20 °C below modern conditions^2^, promoting the formation of continuous and impermeable permafrost regions called yedoma^3^. Yedoma formed during the most recent glacial period (the Last Glacial Maximum, LGM) at ca. 19 ka BP, still covers vast parts of Russian Siberia, Northern Canada and Alaska, and is known to sequester large amounts of organic carbon^4,5^. Organic carbon is stored inside an, up to 1000 m thick, yedoma permafrost or even deeper, in the form of free gas and gas hydrate^6^ – ice-like compounds of water and gas, mainly methane, which are stable under high pressure and low temperature conditions^7^. An important signature of modern yedoma is thermokarst, amalgamated thawed wedges within permafrost^8,9^. Thermokarst is presently ubiquitous in the Arctic and started to form during the warm post-LGM period creating characteristic landforms such as lakes and ravines. Permafrost thawing and thermokarst expansion under accelerated Arctic warming has raised concerns that part of the organic carbon pool stored in such systems is being released into the atmosphere^8,9^. Such processes can be recognized by intensification of thermokarst development in the uppermost permafrost layers^8,10,11^, or by massive gas blowouts, such as those leading to the recent formation of several 60-m deep craters on Yamal Peninsula^12,13^ (Fig. 1). Drilling data, remote-sensing methods and modeling have shown that Arctic permafrost also exists offshore, occupying vast areas of Arctic shelves. These subsea permafrost complexes are also suggested to be thawing under recent warming, causing increased methane gas release from the seabed into the water column, as documented on the East Siberian Arctic Shelf^14^ and west of the Yamal peninsula^15,16^.

In this study we present the first evidence for >280 buried ancient thermokarst structures in the South Kara Sea (~50–250 m water depth; Fig. 1). These are clearly visible on grids of high-resolution reflection seismic data as U-shaped structures within sedimentary bedrock, and lead us to propose the existence of paleo-thermokarst fields with distinctly varied appearance and orientation. They are mapped in several seismic units, indicating heterochronous permafrost thawing processes during the Quaternary. The documentation of submerged yedoma complexes, comprising ancient analogs for modern Pan-Arctic thermokarst, is important as it (1) reveals the geological evolution of ancient thermokarst bodies, (2) draws similarities between modern and ancient thermokarst carbon-storage potential, and (3) demonstrates their sensitivity to environmental changes such as sea level and ocean temperature. We calculate the minimum volume of ancient thermokarst reservoirs, based on their geometry obtained from the seismic data, and estimate the potential amount of carbon stored in these buried thermokarst during past climate warmings. Furthermore, gas hydrae stability modeling, constrained by geochemical analyses and temperature measurements, demonstrates that some of these subsea thermokarst regions presently exist within the gas hydrate stability zone (GHSZ).

## Geology of the South Kara Sea

The South Kara Sea is underlain by 5–10 km thick hydrocarbon-prone Jurassic-Cretaceous bedrock^17^. These deep hydrocarbon provinces are a source of thermogenic gas and locally fluid migration pathways allow the migration of gas from bedrock to the base of the yedoma and/or offshore permafrost regions. High-resolution seismic lines from the South Kara Sea show a variety of seismic amplitude anomalies in the shallow sub seabed, indicating the accumulation of gas in shallow marine sediments^16,18,19^. Drilling results from the Kara Sea^19,20^ show a distinct angular unconformity that separates sedimentary bedrock of Cretaceous – Paleogene age (<145 Ma and >23 Ma) from overlying unlithified marine clays and glacigenic deposits of Quaternary age (<2.6 Ma). This unconformity can be correlated with the Upper Regional Unconformity (URU) of the Barents Sea^21^, formed during repeated advances and retreats of the Barents Sea ice sheet during the Quaternary. In the Kara Sea, it is unknown whether Early and mid-Pleistocene (~2.5 Ma-160 ka) ice sheets extended over the shelf, however Late Saalian (160–140 ka) and Early Weichselian (~90 ka) ice sheets reconstructions predict extended ice cover over the entire study area^22^. During the LGM, glacial ice reached only the eastern flank of East-Prinovozemelsky Trough (Fig. 1), with the remaining shelf and Yamal peninsula subaerial^23,24^. Such conditions would have favored continuous freezing of shelf sediments^1^, promoting deep permafrost development.

### Seismic stratigraphy and evidence for U-shaped thermokarst in bedrock

**Figure 2:**
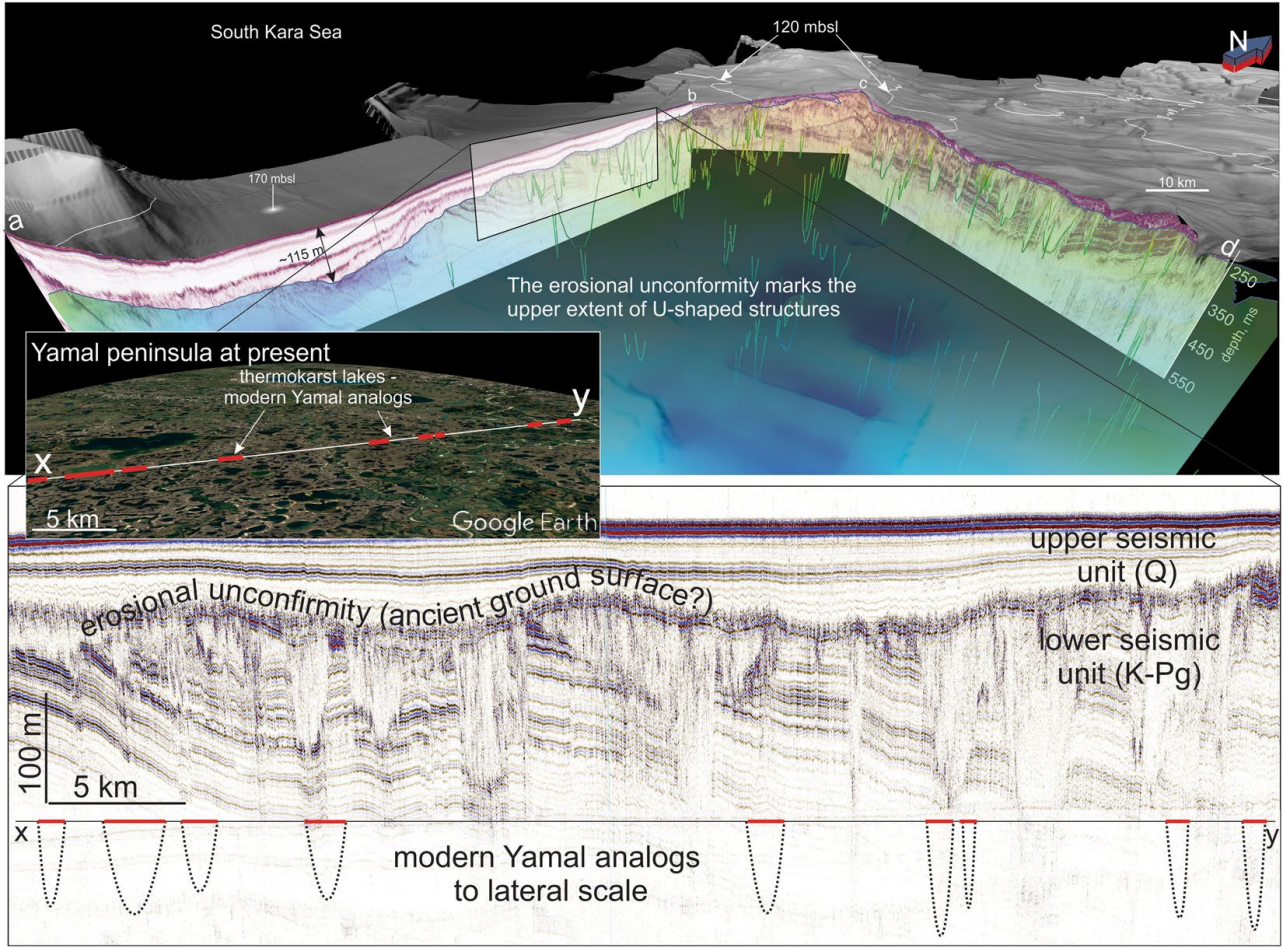
3-D image of the upper sediment cover of the South Kara Sea (see location a-b-c-d in Fig. 1). Shaded gray surface shows high-resolution seismic-derived bathymetry; colored semi-transparent surface indicates unconformity between lower and upper seismic units on top of U-shaped structures (green curves) – remnants of ancient thermokarst. Insets demonstrate the spatial and dimensional similarities between U-shaped structures offshore (middle section) and modern thermokarst lakes onshore Yamal Peninsula (bottom section), which are shown at the same lateral scale. Note different vertical scales (ms and meters); conversion was based on 1600 m/s for acoustic velocity in silt. Satellite image source: “Yamal Peninsula”. 68°55′53 N and 69°24′21 E, eye altitude 12 000 m. Google Earth. December, 2016.

Over 4000 km of high resolution seismic lines collected by MAGE-Marine Arctic Geological Expedition under the Russian Federal Geological Mapping Program 2005–2006^25^ are used in this study. These datasets were acquired with a central signal frequency of ~250 Hz, providing horizontal and vertical resolutions of approximately 30–50 m and 5–7 m, respectively, and a sub-seabed penetration of up to 200 m (using 1600 m/s for acoustic velocity in silts^26^). Seismic stratigraphy reveals two major sediment units here referred to as the upper and lower units. These units are separated by a characteristic angular unconformity (Fig. 2).

**Figure 3:**
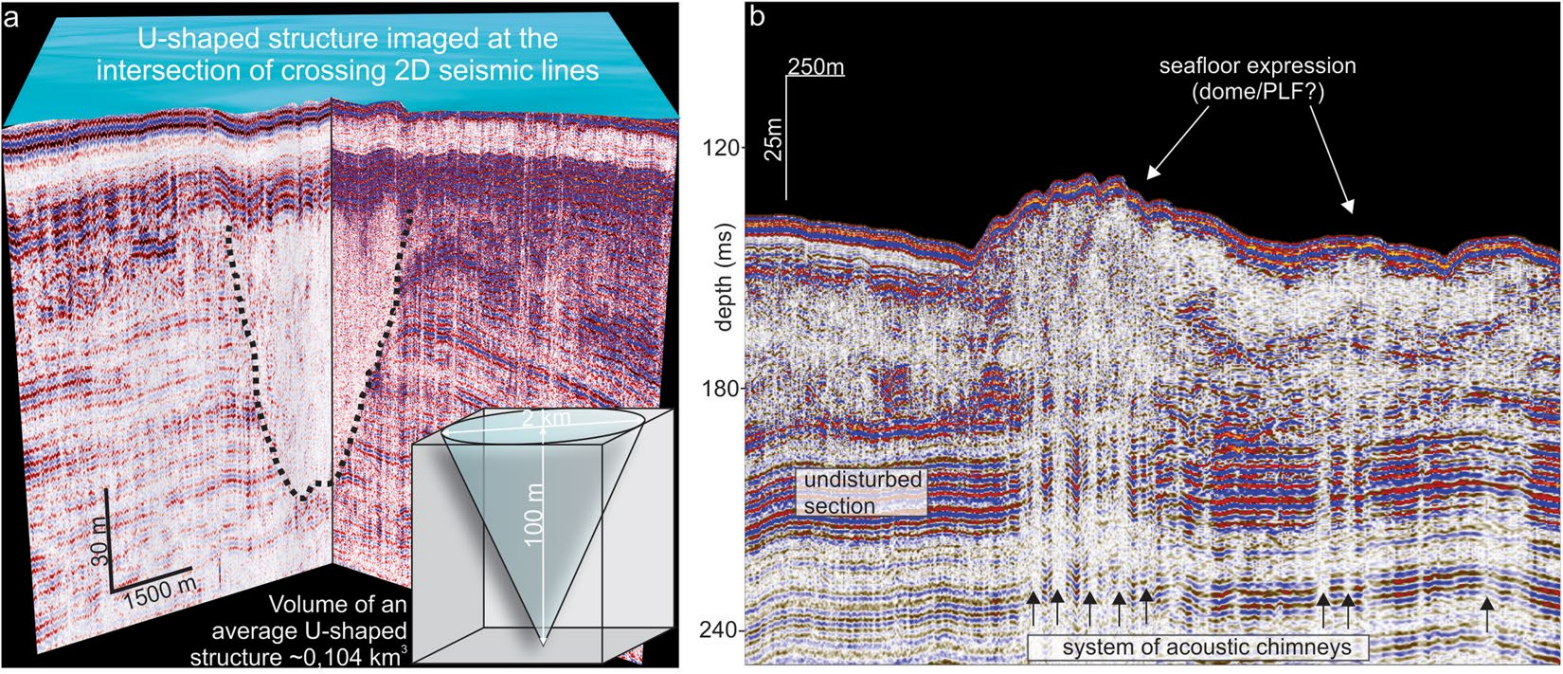
(**a**) Single U-shaped structure in the intersection of two high-resolution seismic lines. Inset shows volumetric approximation for an average-size U-shaped structure, providing constrains for the total thermokarst pool volume within the study area. See location in Fig. 1. (**b**) Fragment of a high-resolution seismic line, showing acoustic chimneys – evidence for shallow fluid flow and gas migration in the South Kara Sea and related seafloor mounds above the chimney system.

The lower unit shows a characteristic pattern of oblique high-amplitude parallel seismic reflections, folded in synclines and anticlines (Fig. 2). It is also characterized by U-shaped incisions with an acoustically transparent internal reflection pattern (Figs 2 and 3a). We call these structures U-shaped because of their clear appearance on the vertically exaggerated seismic profiles, yet in the actual 1:1 scale, they would show up as flattened dished shapes. These U-shaped structures are typically 0.5–5 km wide, up to 80 m deep and appear across the entire study area. They are generally larger and more abundant in today’s shallower water depths (~150–100 mbsl). The major unconformity (separating the upper and lower seismic units) marks their upper extent (Figs 2 and S1a). Beneath the U-shaped features, the bedrock reflections of the lower seismic unit are generally undisturbed but slightly concave. However, some of the U-shaped structures extend downwards as vertical zones with amplitude blanking, masking underlying strata. We also identify seismic amplitude and velocity anomalies, including vertical areas of amplitude blanking (seismic chimneys) (Fig. 3b) and bright spots, seismic reflections with anomalously high amplitude. These anomalies are distributed regardless of any spatial or seismo-stratigraphic pattern, and in some cases, are associated with distinctive doming seafloor features resembling pingo-like features (PLF)^18,27^ (Fig. 3b).

The upper seismic unit comprises a set of distinct sub-horizontal reflections, gradually thinning and cropping out towards the NE (Fig. 2). The thickness of this unit varies significantly, from a maximum of ~135 m in the SW of the study area to less than 60 m across the rest of the South Kara Sea (Fig. S1). By correlation with previous work from the area^20^, sediments of the lower seismic unit are likely of Cretaceous-Paleogene age, while the upper unit comprises mainly Plio-Pleistocene and/or Holocene deposits.

More than 280 buried U-shaped structures exist within an area of at least 32 000 km^2^ in the South Kara Sea, all appearing in the lower seismic unit of Cretaceous-Paleogene sedimentary bedrock. U-shaped structures have been previously identified in lower-resolution seismic data from the Kara Sea^19,20^. Interpretation of these features was highly speculative, given the insufficient data, but authors favored river paleo-channels and/or permafrost-bounded shallow gas accumulations^19,25^. Yet, the distinct undisturbed reflections observed beneath these structures and their dished shapes are not typical of gas-related features, which are more commonly associated with vertical zones of amplitude blanking or bright spots^28^.The primarily erosive processes related to paleo-river channels or elongated gullies may better explain the observed truncated layering of the bedrock, however, most of the U-shaped structures located at the intersections of crossing seismic lines show confined isometric “dished shapes” (Fig. 3a). Additionally, even large U-shaped structures can not be traced on neighboring seismic lines, which also contradicts a hypothesis of elongated paleo-channels. We therefore find existing explanations for the origin of the buried U-shaped structures lacking, and seek an alternative.

The lateral scale and spatial distribution of the observed U-shaped structures bear a striking resemblance to modern thermokarst lakes on the Yamal peninsula and Ural coast^12,13^ just a few tens of kilometers away from our study area (insets of Fig. 2). The U-shaped structures mapped in the seismic data are on average much deeper (<100 m) than the modern subaerial thermokarst lakes (<30 m)^8,9^, however this may be because modern thermokarst lakes merely represent the upper part of thermokarst formations, while the seismic data reveal the entire depth of paleo-thermokarst build-ups. Therefore, we hypothesize that the widespread U-shaped structures in the lower seismic unit are remnants of thermokarst landscapes. Under many of the U-shaped structures, reflections become concave, likely indicating anomalous low-velocity zones. A low velocity zone may stem from a particular sediment type infill, and/or the presence of free gas within the ancient thermokarst^26^ (inset of Fig. 2). Given that thermokast formation is usually associated with interglacials, where rising temperatures promote permafrost thawing, and that the mapped U-shaped features are restricted to the lower seismic unit which is overlain by mainly Plio-Pleistocene and/or Holocene deposits, we infer that the identified thermokarst landscape developed during pre-LGM interglacial(s).

The timeframe for the generation and modification of thermokarst structures within thawing permafrost ranges widely from several thousand years^9,29^ to less than one year^13^ as exemplified by the recent formation of a ~60 m deep thermokarst crater onshore Yamal Peninsula. Such enormous, near-instantaneous events, may occur in terrestrial Arctic regions as increasingly higher summer temperatures trigger rapid outbursts of gas from permafrost/gas hydrate-bearing formations. It is possible that a similar process led to the formation of some of the U-shaped structures in the South Kara Sea. A time window for such thermokarst generation can be inferred from existing chronostratigraphic analyses of the Kara Sea^19,20^. This reveals that relict thermokarst is imbedded in Paleogene (Cretaceous? -Paleogene) deposits of the lower complex (Fig. 2). The unconformity between the lower and upper complexes marks ancient land surfaces and serves as an important event-boundary created by a distinct time period of non-sedimentation (hiatus) in Paleogene-Neogene sediments^20^. Glacial sedimentation and erosion subsequently took place in the Quaternary. We suggest that the first generation of thermokarst started to form during the Late Neogene – Early Quaternary time, when the region experienced a major sea level low stand accompanied by subaerial exposure and denudation^20^. Because many U-shaped structures appear also under the most submerged parts of the upper regional unconformity, a significant sea level regression must have reached at least ~280 mbsl (Fig. S1). Such a significant change in the relative sea level indicates that the South Kara Sea experienced a regional high-amplitude tectonic uplift. This is in good agreement with a previously proposed model of neo-tectonic movements, which suggests that uplift reached +250–500 m in the Kara Sea during the Neogene - Early Quaternary^20^. During the Plio-Pleistocene, tectonic subsidence and concomitant sea level rise resulted in the gradual inundation of subaerial regions, including thermokarst, and a transition into a marine depositional environment. Finally, deposition of Quaternary marine and glacial-marine facies created a sediment blanket on top of the U-shaped structures. Due to the transgressive nature of the sea level rise, U-shaped structures were buried at different times, since shallower areas of the ancient ground surface were inundated much later than deeper areas and thus had a longer exposure time. This is consistent with both the greater depths and denser distribution of thermokarst in the shallower N-NW part of the study area (Figs 2 and S1a).

The shallowest region studied, with water depths <120 mbsl (max. level of Late Pleistocene sea regressions), shows a complicated sub-seafloor stratigraphy, with structures marked by active erosion during the LGM. Glacial-marine complexes, likely represented by a terminal moraine pinch (Fig. 1), occasionally leave <10 m thick Holocene cover on top of the bedrock (Figs 2 and S1). Single relict thermokarst structures cut through the upper seismic unit and show a morphological expression on the seafloor as a local depression. This either indicates that the thermokarst structures here are of a very young age, and/or erosion has taken in the overlying sediment section. Age dating of sediments from U-shaped structures in the future, coupled with detailed seismo-stratigraphic reconstructions, would shed more light on the timing of such geological processes and their interaction with glaciations and eustatic sea level variations.

### Ancient thermokarst carbon storage and its vulnerability to ocean warming

Various approaches may be taken to estimate carbon storage within the mapped paleo-thermokarst. The modern North Siberian, yedoma covers an area of ~1 million km^2^ and its carbon pool is estimated to be up to ~500 Gt^4^, comparable to cumulative anthropogenic carbon emissions from 1870 to the present^30^. Of this, a disproportionally large volume (approximately half) is stored within thermokast^8^. Assuming a similar density of thermokarst in the South Kara Sea and the modern North Siberian yedoma region, the carbon pool in our study area (32 000 km^2^) may reach a maximum of ~8 Gt. Alternatively, the carbon pool may be approximated assuming a carbon content of 2–5% by mass, as previously defined for modern yedoma regions in North Siberia and Alaska^9^. Using a minimum estimate of the mapped volume of subsea thermokarst within our study area of approximately 28 km^3^ (volume of one medium size U-shaped structure multiplied by their total number of 280, Fig. 3a), and an average thermokarst sediment density of 1800 kg m^−3^, the resulting carbon content in our study area varies from 1 Gt (2% carbon by mass) to 2.5 Gt (5% carbon by mass). The actual volume may be larger, because of the unaccounted thermokarst located between seismic lines. For comparison, the calculated volume represents approximately a third of the reservoir rock volume of Snøhvit, one of the largest discovered gas fields in the Norwegian Barents Sea (approx., 89 km^3^)^31^. Finally, the most conservative carbon stock estimates for our study area can be derived based on the carbon content of deep, drained thermokarst-lake basins in Siberia^9^ of Holocene age. Here, carbon content varies from 10 to 50 kg m^−3^ in the upper 10 meters of the sediment section, depending on depth and thermokarst facies type. Based on these numbers, the carbon estimates for buried subsea thermokarst in our study area may vary from 0.5 to 2.2 Gt, which is within the minimum and maximum calculated range, i.e. from 0.5 to 8 Gt.

The sub-seabed of the South Kara Sea lacks a thick Quaternary sediment seal, and over the shallower NE region (<~150 mbsl) this seal thins to <10 m. Yet, methane concentrations in sediments from sediment cores analyzed from >100 grid-spaced gas-sampling stations in the South Kara Sea show only moderate levels, with maximum and average values of 734.33 and 16.35 nl/g respectively^25^. These low values may be explained by heterogeneous and rapid depletion of methane in the shallow sediments, or simply a lack of sampling stations above the U-shape structures. Our estimates suggest that ancient thermokarst has the volume capacity to store significant amounts of methane, yet assessment of its actual hydrocarbon potential requires target- oriented drilling and sampling.

The stability of vast thermokarst carbon storage regions in the Arctic is a matter of concern given present atmospheric and ocean warming scenarios, which are promoting thawing of permafrost and gas hydrate dissociation both onshore and offshore across shallow Arctic shelves, such as the East Siberian Arctic and the Beaufort Sea Shelves^27,32,33^. Most thermokarst structures in the South Kara Sea are located in water depths >50 m, beyond the limits of continuous permafrost distribution^34^, suggesting that the majority of the mapped ancient thermokarst may not be vulnerable to ocean warming. Yet, in the deepest part of the study area, the South Kara Sea depression, methane stored within the thermokarst can be preserved in the form of gas hydrate, which is highly sensitive to changes in temperature and pressure.

### Modeling of the gas hydrate stability zone in the South Kara Sea

**Figure 4:**
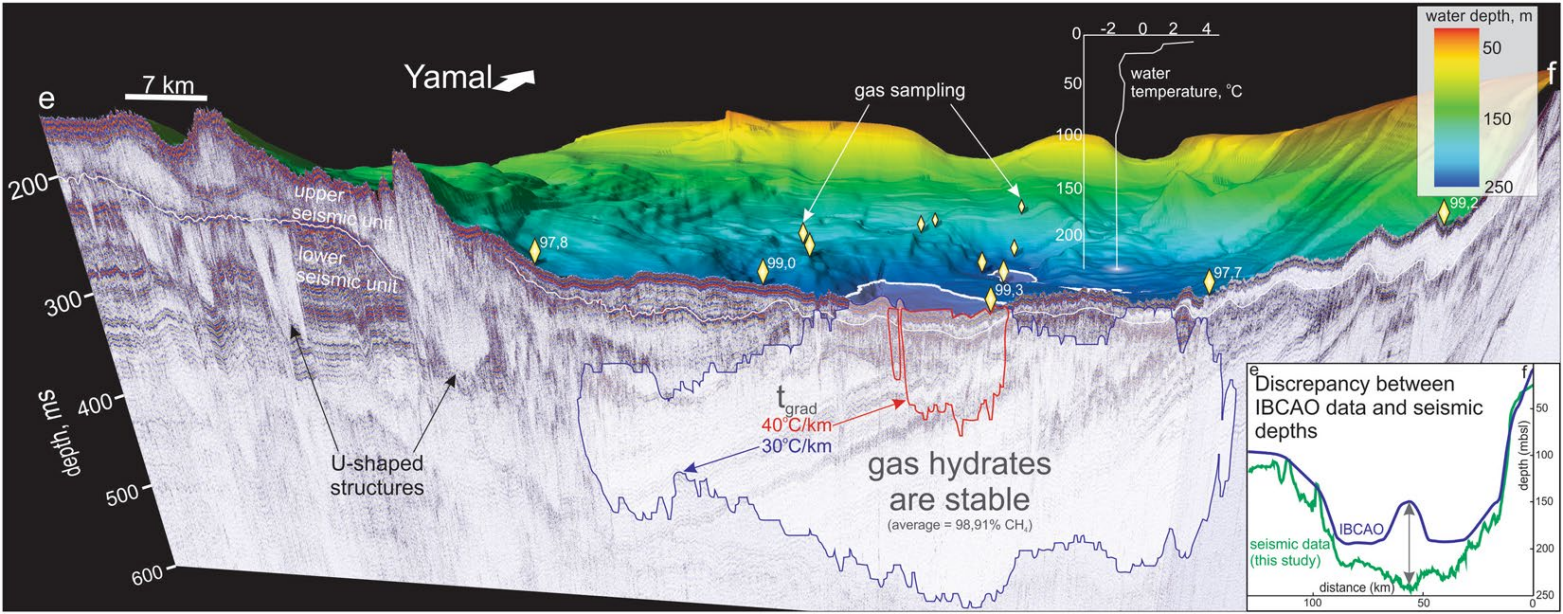
Fragment of a seismic line (see location e-f in Fig. 1) along which we implemented gas hydrate modeling for 30 and 40 °C/km geothermal gradients. Yellow diamonds indicate gas sampling stations. Our results show that approximately 20% of mapped ancient thermokarst may presently exist within the gas hydrate stability zone.

Methane gas in ancient subsea thermokarst may exist dissolved in the fluids, in the free gas phase or in the form of gas hydrate. Gas hydrate within permafrost has been repeatedly drilled on Yamal Peninsula, e.g. in permafrost regions above the Bovanenkovo gas/gas condensate field^35^, yet, there is no publically available data on offshore gas hydrate from drilling in the South Kara Sea. Based on our gas chromatography analyses, bottom water temperature measurements and precise water depths we have modeled the present-day GHSZ in the South Kara Sea. This confirms that the extremely low bottom water temperatures (−1.8 °C) favor conditions for gas hydrate generation under the relatively shallow Kara Sea shelf (Fig. 4).

Our new regional-scale seismic-based bathymetry represents a significant improvement in accuracy and vertical resolution compared to previously available IBCAO data^36^ (Fig. 1, inset of Fig. 4). For example, IBCAO data underestimated water depths by up to 80 m in the South Kara Sea depression (Fig. 4), which significantly changes the result of GHSZ modeling. Present-day South Kara Sea bottom water temperatures are as low as −1.8 °C in water depths >100 m, and show no seasonal changes across the study area^37^ (Fig. 4). Therefore, we used −1.8 °C for the GHSZ modeling along a selected seismic profile, considered representative of the study area given that it crosses the entire South Kara Sea depression at ~180−~250 m water depths (Figs 1 and 4). Gas chromatography analyses from 17 bottom sediment gas-sampling stations located on or close to the modeling profile (Figs 1 and 4, methods), show methane gas concentration variations from 97.72 to 99.82%, with an average of 98.91%^25^. Large uncertainties exist in the geothermal data from the South Kara Sea and West Siberian Basin, mainly due to the lack of reliable measurements offshore. We therefore modeled the GHSZ for two scenarios – one for 30 and the other one for 40 °C/km temperature gradients (the most realistic numbers based on the existing literature^38,39^). Water depths along the modeling profile exceed 120 m, thus there is no sub-sea permafrost and no related sub-seabed temperature cooling. Using the 30 °C/km gradient, a more than 270 m thick GHSZ is predicted in ~220–250 m water depths. This GHSZ can extend over max. ~50 km along the selected profile (Figs 1 and 4). Thinning of the GHSZ occurs towards shallower water depths, where it outcrops at ~220 mbsl (Fig. 4). Generally, the GHSZ does not reach the seafloor so that both the upper and lower GHSZ boundaries are located in the sub seabed. An exception is the narrow deeper basin (~235–250 mbsl), where the upper boundary of the GHSZ outcrops at the seafloor over ~10 km, potentially exposing any gas hydrate bearing sediments close to the seafloor (Fig. 4). Such a GHSZ configuration, to our knowledge, has never been observed elsewhere. Our gas hydrate modeling shows that up to ~20% of the observed ancient thermokarst are currently within the GHSZ (Fig. 4). However, during glacial-interglacial periods, changes in environmental conditions across the South Kara Sea shelf: from subaerial, to interchanging “cold” subglacial/subsea conditions and consequently to present day “warm” subsea conditions (Fig. S2), would have led to repeated expansions and contractions of the GHSZ, potentially associated with methane release. The modern hydrologic regime of the South Kara Sea shows constant water temperature at depths >100 mbsl, with no observed influence from warmer deep Atlantic or Barents Sea currents at such depths^37^. The current GHSZ in the South Kara Sea can therefore be considered stable, yet remains highly sensitive to any potential changes in the future.

## Conclusions

- High-resolution seismic data over a 32 000 km^2^ wide area in the South Kara Sea document the existence of more than 280 U-shaped thermokarst structures. These characteristic features appear in Cretaceous-Paleogene sedimentary bedrock, below overlying silty marine clays and glacial sediments.
- Single U-shaped structures are semi-circular, up to several km wide, 80 m deep and bear a striking resemblance to modern onshore thermokarst craters in the Arctic.
- U-shaped structures, most likely generated during various interglacial stages of Plio-Pleistocene time, are interpreted to be buried ancient thermokarst formations.
- The ancient thermokarst may, by analogue to modern yedoma, represent a carbon pool exceeding 28 km^3^ total volume, with an estimated carbon content ranging from approximately 0.5 to 8 Gt within our study area.
- Modeling shows that approximately 20% of the mapped ancient thermokarst is located within the current GHSZ.
- Ancient thermokarst regions may exist in vast regions of Arctic continental shelves presenting potential, and yet unexplored, carbon storage pools.

## Methods

### Gas Hydrate Stability Zone Modeling

The thickness of the gas hydrate stability zone is estimated using the CSMHYD program’s P-T phase boundary curves for hydrates with mixed gas compositions^40^. The program uses an algorithm based on Gibbs energy minimization and calculates multiphase equilibria for any given temperature or pressure. The program was executed such as it calculated pressure at which hydrates are stable for given temperature values. The pressure estimates were then converted to depths using the density of seawater (1027 kg m^−3^) and acceleration due to gravity (9.8 m s^−2^). Pressure was assumed to be hydrostatic and a pore-water salinity of 35 g l^−1^ was used in our models. The resulting theoretical temperature-depth profile for a given gas composition was compared with the thermal profile from CTD data and geothermal gradient (assuming a linear gradient at 1 m vertical resolution) at each location (~100 m spatial interval) along the seismic transect. If the temperature from the theoretical profile at a specific depth was greater than that from field data, hydrates were deemed to be stable at that depth.

### Sedimentary Gas Extraction

Interstitial gas samples were extracted with the degassing set SUOK-DG (Patent (19) RU (11) 2348931 (13) C1) including centrifugal pump, supersonic ejector and hollow stainless steel working volume. Pre-weighted sediment samples were loaded into the sampling chamber and completely re-suspended by degassed water under high pressure. Interstitial gas phase was separated from the homogenous sediment pulp by supersonic ejector and fed into the graded volume meter. After the volume recording, the isolated sedimentary gas was transferred into the clean crimped vials with butyl-rubber stoppers.

### Determination of methane and C_2_–C_5_ hydrocarbon gases

Hydrocarbon gases composition (C_1_–C_4_) was analyzed using Shimadzu 2014 gas chromatograph equipped with flame ionization detector and Restek Rt-Aluminia BOND/Na_2_SO_4_ wide-bore capillary column (i.d. 0,53 mm, length 50 m, film thickness 10 µm) attached to the packed injector. Helium was used as a carrier gas at a flow rate 25 ml/min. Certified gas mixtures were used as external standards. The detection limit of the analysis was 50 ppb, for CH_4_ the error of instrumental measurements did not exceed 5%.

## Electronic supplementary material

Supplementary material

## Data Availability

The seismic and geochemical datasets analyzed during the current study are available from the corresponding author on reasonable request.
