# Supplementary material for: Shallow carbon storage in ancient buried thermokarst in the South Kara Sea
Source: Sci Rep. 2018 Sep 25;8:14342. doi: 10.1038/s41598-018-32826-z (PMC6156565; doi:10.1038/s41598-018-32826-z)
Supplement: Supplementary file 1 — Supplementary material [file 41598_2018_32826_MOESM1_ESM.pdf]

Title:

Shallow carbon storage in ancient buried thermokarst in the South Kara Sea

Author list and affiliations

Alexey Portnov [1,2*], Jurgen Mienert [2], Monica Winsborrow [2], Karin Andreassen [2], Sunil Vadakkepuliyambatta [2], Peter Semenov [3], Valery Gataullin [4]

[1] *School of Earth Sciences, The Ohio State University, Columbus, Ohio, USA*

[2] *CAGE - Centre for Arctic Gas Hydrate, Environment and Climate, Department of Geoscience, UiT The Arctic University of Norway, 9037 Tromsø, Norway*

[3] *FSBI VNIIOkeangeologia, Saint-Petersburg, Russia*

[4] *7159 Crofton Court, Reynoldsburg, OH 43068*

*Correspondence to:

Alexey Portnov

Email: portnovalexey@gmail.com

Phone: +1 614 7952683; +47 93944051

Present postal address:

School of Earth Sciences,

The Ohio State University,

385 Mendenhall

Supplementary material

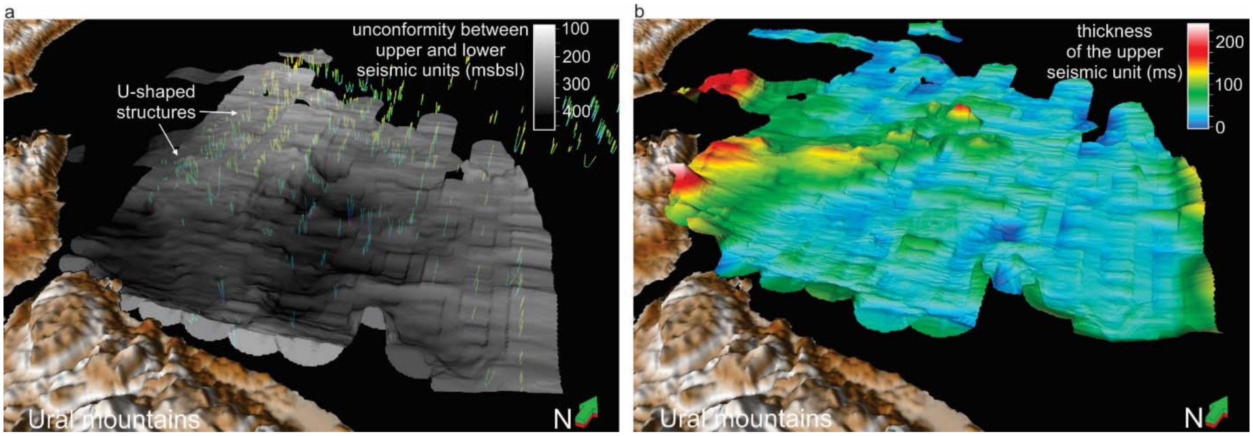

Supplementary Figure 1. (a) Gray shaded surface shows unconformity between upper and lower seismic units (TWT-milliseconds below the sea level). Colored curves show interpreted U-shaped structures along the high-resolution seismic lines. (b) Thickness of the upper seismic unit (TWT-milliseconds).

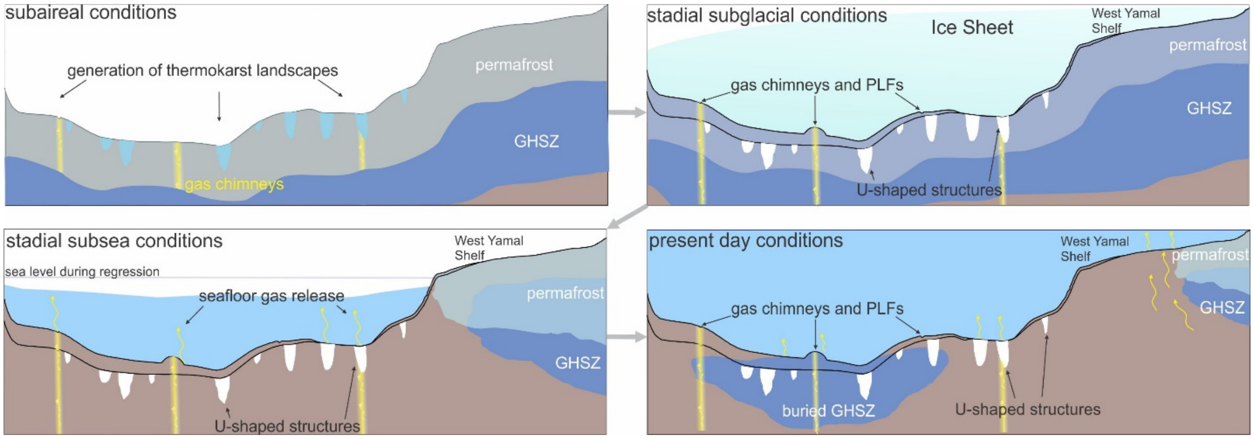

Supplementary Figure 2. Inferred changes in environmental conditions during Quaternary and their potential effect on ancient thermokarst carbon pool in the South Kara Sea: from subaerial, to interchanging “cold” subglacial/subsea conditions and consequently to present day “warm” subsea conditions.
